# Magma transport in sheet intrusions of the Alnö carbonatite complex, central Sweden

**DOI:** 10.1038/srep27635

**Published:** 2016-06-10

**Authors:** Magnus Andersson, Bjarne S. G. Almqvist, Steffi Burchardt, Valentin R. Troll, Alireza Malehmir, Ian Snowball, Lutz Kübler

**Affiliations:** 1 Department of Earth Sciences, Uppsala University, Uppsala, Sweden; 2 Geological Survey of Sweden, Uppsala, Sweden

## Abstract

Magma transport through the Earth’s crust occurs dominantly via sheet intrusions, such as dykes and cone-sheets, and is fundamental to crustal evolution, volcanic eruptions and geochemical element cycling. However, reliable methods to reconstruct flow direction in solidified sheet intrusions have proved elusive. Anisotropy of magnetic susceptibility (AMS) in magmatic sheets is often interpreted as primary magma flow, but magnetic fabrics can be modified by post-emplacement processes, making interpretation of AMS data ambiguous. Here we present AMS data from cone-sheets in the Alnö carbonatite complex, central Sweden. We discuss six scenarios of syn- and post-emplacement processes that can modify AMS fabrics and offer a conceptual framework for systematic interpretation of magma movements in sheet intrusions. The AMS fabrics in the Alnö cone-sheets are dominantly oblate with magnetic foliations parallel to sheet orientations. These fabrics may result from primary lateral flow or from sheet closure at the terminal stage of magma transport. As the cone-sheets are discontinuous along their strike direction, sheet closure is the most probable process to explain the observed AMS fabrics. We argue that these fabrics may be common to cone-sheets and an integrated geology, petrology and AMS approach can be used to distinguish them from primary flow fabrics.

Within many central volcanoes, cone-sheet and dyke intrusions represent the main channels of magma transport from magma reservoirs to the Earth’s surface. To fully capitalise on the information contained in solidified sheet intrusions, which can improve risk assessment in volcanically active areas or be used to locate sources of mineral-bearing intrusions in exploration efforts, a comprehensive understanding of intra-sheet processes is desirable. A series of workers have used AMS to determine the magmatic flow direction and the internal flow dynamics of magmatic sheet intrusions^1^,^2^,^3^,^4^,^5^,^6^. A recent study on AMS in cone-sheets of the Ardnamurchan central complex in northwestern Scotland, for example, observed lateral flow patterns in the majority of concentric cone-sheets^7^, which led the authors to propose a lateral magma transport model on a regional scale as mode of emplacement for the Ardnamurchan cone-sheets. However, 3D projections of the same sheets indicated a magma source directly beneath the Ardnamurchan volcano^8^, and moreover, flow patterns frequently diverged from the overall flow direction of these intrusions^9^,^10^. An improved understanding of the origin of magnetic susceptibility fabrics in magmatic sheet intrusions is, therefore, crucial if AMS is to be used as a reliable tool to assess flow direction and flow dynamics in intrusive sheets.

The Alnö ring complex in central Sweden is a subvolcanic carbonatite and alkaline igneous complex that hosts numerous cone-sheets and dyke intrusions^11^,^12^,^13^,^14^,^15^. Here we present the results of a systematic AMS study of twelve carbonatite sheet localities at Alnö and discuss the AMS data in the context of magma flow dynamics. We build upon earlier geological studies at Alnö that inferred a former feeder reservoir, or magma chamber, at 1 to 4 km depth beneath the present-day landsurface^11^,^12^,^13^,^14^,^15^ and thus aim to improve our understanding of near-surface magma transport in magmatic sheet intrusions. Since magnetite is one of the first minerals to crystallise from a cooling carbonatite magma, the magnetic fabric in carbonatite sheet intrusions should record, in principle, similar processes to sheet intrusions that form from silicate magmas with higher viscosity^16^.

## Magnetic Anisotropy Associated with Magma Flow

Magnetic susceptibility is a symmetric second rank tensor that is conventionally represented by three orthogonal principal axes, maximum (*k*_*1*_), intermediate (*k*_*2*_), and minimum (*k*_*3*_) susceptibility, which are usually illustrated as an ellipsoid. A wide range of parameters to quantify AMS can be determined (e.g.^17^,^18^,^19^,^20^), and we focus on bulk magnetic susceptibility (*K*_*m*_), degree of anisotropy (*P*_*j*_), and shape factor (*T*), as they are commonly employed parameters to describe magnetic anistropy^17^,^19^. *P*_*j*_ is equal to or larger than 1 and describes the size of the magnetic anisotropy (*P*_*j*_ = 1.0 for isotropic materials)^17^. *T* describes the shape of the susceptibility ellipsoid and ranges from +1 (oblate) to −1 (prolate)^17^. In addition, we use magnetic hysteresis parameters and coercivity of remanence to determine the magnetic domain state that controls the magnetic susceptibility. When laminar magma flow occurs in a magmatic sheet intrusion, it exerts a shear force that tends to orient prismatic and tabular mineral grains parallel to the flow direction, with often only a small angular deviation between the long axis of the grain and the flow direction^2^. The main advantage of AMS compared to traditional textural and microstructural techniques is that a large number of samples can rapidly be measured and provide an accurate determination of the overall orientation of the sum of all mineral grains in each sample, although it is naturally biased towards minerals that have high magnetic susceptibility^20^.

**Figure 1:**
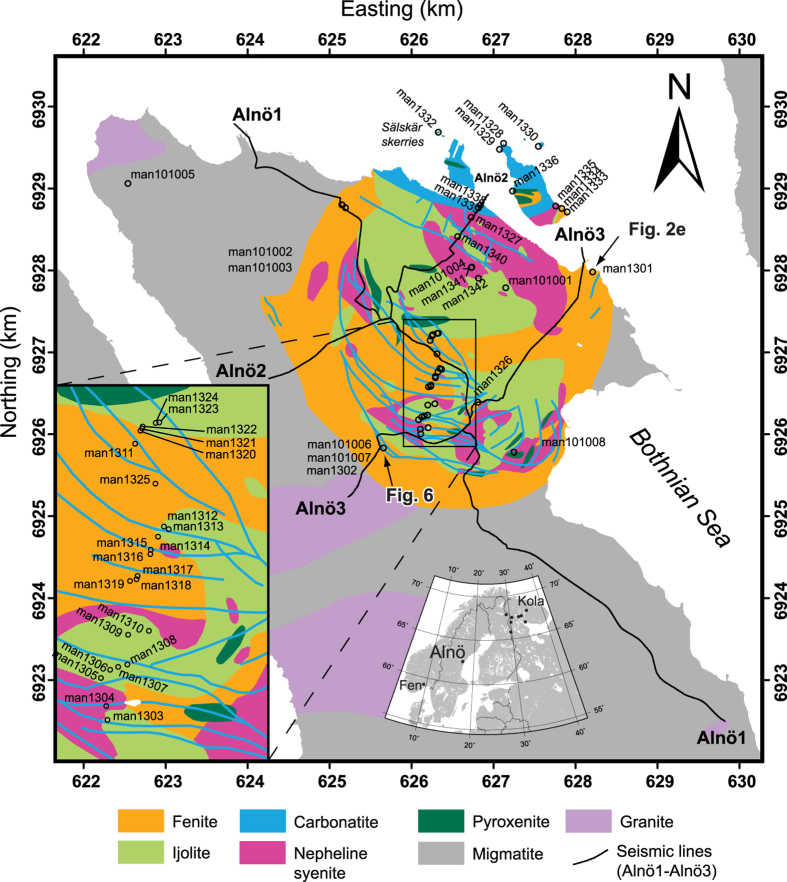
Geological map of the Alnö alkaline and carbonatite ring complex. Oriented drill-cores were sampled along a north-south profile across the central part of the ring complex (sample localities are marked with respective sample numbers) and a magnification of this central area is shown in the lower left side. Key-outcrops in the ring complex were sampled in detail to investigate outcrop-scale variations in magnetic anisotropy. Black lines show the available reflection seismic profiles (Alnö1, 2, and 3) in the study area^15^. Inset map in the bottom centre shows northern Europe and the locations of Alnö in central Sweden, the coeval Fen complex in Norway, and the alkaline and carbonatite intrusions in northern Finland and northwestern Russia. The geological map was kindly provided by © Geological Survey of Sweden and prepared in the Q-GIS open source software and Adobe Illustrator CS4. The coordinate system is SWEREF99 TM (UTM zone 33N).

Pioneered by Khan^1^, AMS has been used in many petrofabric studies of igneous rocks to determine magma flow directions, but the method itself and its applications to igneous rocks is continuously being refined (see e.g. review by Cañón-Tapia^21^). From theoretical considerations, Khan^1^ argued that *k*_*2*_ should coincide with the flow direction. This claim was supported by Ellwood^22^, who interpreted *k*_*2*_ as the flow direction in Icelandic basaltic dykes. Later, the axis defining the magnetic lineation, *k*_*1*_, was used as the main indicator for flow direction, with application to dykes in the Koolau Complex on Oahu (Hawaii)^2^. However, studies of Tertiary dykes in east Greenland demonstrated drawbacks with the approach of using *k*_*1*_, because sub-vertical magma flow was indicated using *k*_*1*_, which deviated strongly from the sub-horizontal flow directions interpreted from outcrop observations^5^. As a consequence, Geoffroy *et al*.^5^ proposed the pole to the magnetic foliation, *k*_*3*_, as a more robust parameter for interpreting magma flow direction. Numerous studies subsequently employed AMS to reveal information on magma flow from the measured magnetic signal, e.g.^1^,^2^,^4^,^9^,^18^,^22^, but the use of different interpretation schemes has led to a muddled view of how best to interpret AMS results. A number of studies have by now indicated that AMS alone might be insufficient to infer flow directions in igneous rocks and should ideally be complemented by other analytical information (e.g. fabric analysis in the field or shape and crystallographically preferred mineral orientation determined by optical or scanning electron microscopy)^5^,^7^,^9^,^23^,^24^,^25^. Some studies emphasise that mineral specific magnetic susceptibility may also play a role and advise to separate different mineral contributions^26^. Here we use an approach that involves detailed sampling and AMS measurements of carbonatite sheets and wall rock across sheet intrusions from Alnö (Fig. 1) which we combine with field observations and microscopic data. In connection with flow-test calculations^2^,^5^ and a compilation of geologically plausible syn- and post-emplacement scenarios^7^,^8^,^9^,^10^,^11^,^12^,^14^,^15^,^27^,^28^, we use the AMS results to explain sheet emplacement in the Alnö ring complex.

## Geological Setting and Petrography

**Figure 2:**
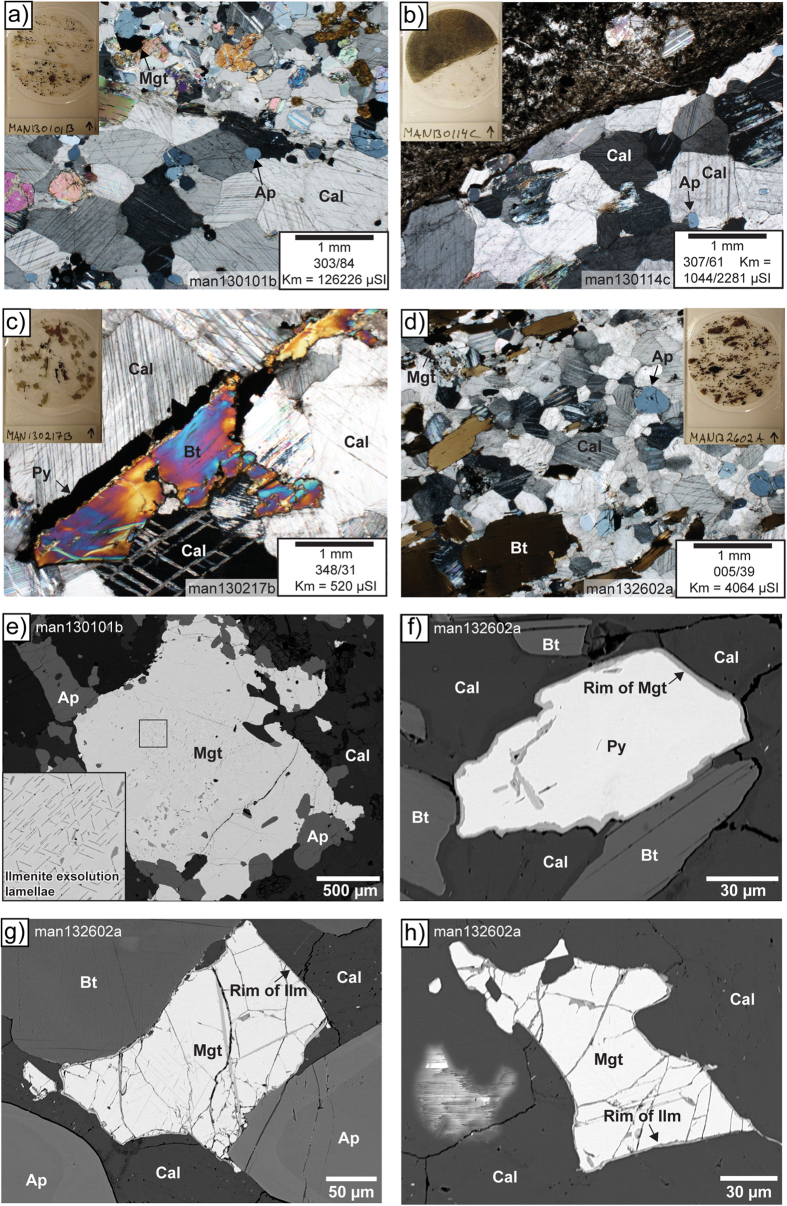
Mineralogy of the carbonatite sheet intrusions. Micro-photographs (**a–d**) and backscattered electron micro-images (**e–h**) from oriented drill core samples of carbonatite sheet intrusions are shown. Inset pictures in a-d show the complete thin section. The thin sections were prepared from cuts perpendicular to the drill direction. Drill direction (azimuth/dip) and *K*_*m*_ are indicated. (**a**) Sample man130101b consists of calcite, apatite, biotite, and magnetite. (**b**) Sample man130114c consists of a coarse-grained carbonatite that contains calcite and apatite in contact with a fine-grained darker carbonatite, which contains calcite, biotite, and minor amounts of opaque minerals; magnetite, pyrite, and pyrrhotite. The higher *K*_*m*_ value is for the coarse-grained carbonatite and the lower for the fine-grained. (**c**) Sample man130217b consists of more coarse-grained minerals compared with the other samples. It contains calcite, biotite, pyrite, pyrrhotite, and minor amount of chalcopyrite. (**d**) Sample man132602a contains fine-grained calcite, magnetite, and pyrite and coarser biotite grains. Subpanels e-h show backscattered electron micro-images: (**e**) large euhedral magnetite grain intergrown with apatite and resting in a matrix of calcite. The inner part of the magnetite grain shows well developed exsolution lamellae of ilmenite, which are magnified in the inset image (man130101b) and which imply slow and static cooling of the mineral. (**f**) Smaller pyrite grain with ~2 μm rim overgrowth of a magnetite rim (man132602a). (**g,h**) Small subhedral magnetite grains within a framework of larger primocrysts, showing a ~2 μm overgrowth rim of mainly ilmenite (man132602a), suggesting that hydrothermal processes and recrystallisation were not significant factors for the ore minerals. Ap: apatite, Bt: biotite, Cal: calcite, Ilm: ilmenite, Mgt: magnetite, and Py: pyrite.

The Alnö ring complex in central Sweden is one of the larger carbonatite and alkaline ring-type intrusions in the world (radius ~2.5 km, Fig. 1)^11^,^12^,^13^ and was emplaced into Palaeoproterozoic migmatitic country rock at 584 ± 7 Ma^29^. A halo of fenite surrounds the intrusion, which resulted from metasomatic alteration of the wall rock by CO_2_-rich solutions from the intruding carbonatites and alkaline silicate rocks^30^. The degree of fenitisation varies with distance from the intrusion. An unaltered migmatite approximately 500 m from the intrusion gives way to a strongly altered variety with no free quartz remaining close to the intrusion^13^,^30^. The Alnö ring complex itself contains a wide variety of igneous lithologies, including an alkaline silicate rock suite (nepheline syenite, ijolite, and pyroxenite) and a range of carbonatite types with variable grain sizes and compositions^13^,^30^,^31^. Carbonatites are named sövite when they consist dominantly of medium to coarse-grained calcite and are then usually snow-white in colour. Carbonatite rocks at Alnö become darker with increasing contents of silicates and oxide minerals and are then called silico-sövite^13^. For the purpose of this study, we use the wider term carbonatite throughout the text for igneous rocks with >50 volume percent of calcite. Calcite grains can exceed 1 mm in size, and crystal borders usually show ~120° triple junctions. This feature is characteristic of equilibrium assemblages, e.g.^32^, indicating that the rock has not been subjected to large post-magmatic differential stresses (Fig. 2a–d). The carbonatites in our sample suite also contain smaller amounts of magnetite, biotite, and apatite (Fig. 2). Magnetite crystals observed in thin sections have grain sizes that vary between 0.1 and 1 mm and are irregular in shape (Fig. 2a,b), whereas apatite crystals range between 0.1–0.5 mm in grain size and have hexagonal to circular crystal outlines in sections perpendicular to their long axis.

The subsurface geology of the Alnö complex was initially inferred from surface geological mapping of dip and dip direction of alkaline silicate and carbonatite sheet intrusions^11^,^12^,^13^. A dome-shaped magma chamber with the roof at ~1.5 km below the present day land surface was suggested to have supplied steeply dipping radial dykes and shallow to moderately dipping carbonatite cone-sheets^12^,^13^,^33^. Recently acquired high-resolution reflection seismic profiles, ground gravity and magnetic measurements indicate that a magma reservoir, up to 1 km thick and possibly saucer-shaped, existed at ~3 km depth below the present day land surface^15^. As erosion has been suggested to have removed between 500 and 2000 m, this pluton would have been originally situated at a depth of 3500 to 5000 m below the land surface at the time of magmatic emplacement^11^,^13^,^15^.

## Results

**Figure 3:**
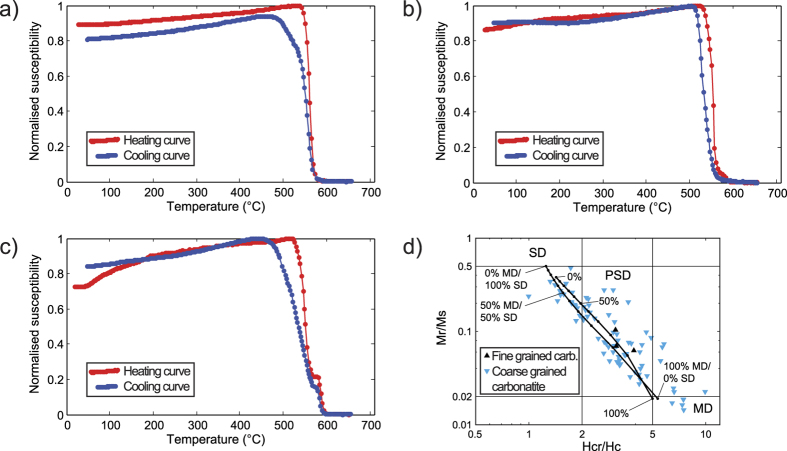
Magnetic susceptibility versus temperature and normalised magnetic hysteresis parameters. (**a–c**) show normalised magnetic susceptibility as a function of temperature for three different carbonatite samples from the locations man1301 (**a**), man1328 (**b**) and man1333 (**c**) (see Fig. 1). The heating path is indicated by the red curve, whereas the cooling path is shown by the blue curve. (**d**) Normalised magnetic hysteresis parameters, with coercivity and magnetisation ratios shown in a ‘Day-plot’^65^. *M*_*s*_: saturation magnetisation; *M*_*r*_: saturation remanent magnetisation; *H*_*c*_: coercivity; *H*_*cr*_: coercivity of remanence. Different fields in the figure refer to the domain state of magnetite, SD: single domain, PSD: pseudo-single domain, MD: multi-domain. Black-dotted lines represent two theoretical mixing curves of SD and MD magnetite in different volume proportions^34^, implying a mixture of SD, PSD, and MD is present at Alnö.

Forty locations were drilled and 219 drill cores were sampled. Just over half of the cores (118) come from twelve carbonatite sheets while the remaining ones are from country rock outcrops. In addition, eight oriented blocks were collected in a pilot study, from which 36 cores were extracted in the laboratory. Magnetic property measurements (see Methods section), including susceptibility as a function of temperature (Fig. 3a–c) and applied field (Fig. 3d), indicate that magnetite is the dominant ferromagnetic mineral (*sensu lato*) in Alnö carbonatite. Curie temperatures for three representative carbonatite samples are between 570 and 580 °C, which is consistent with little or no substitution of iron by titanium in the resident magnetite crystals.

**Figure 4:**
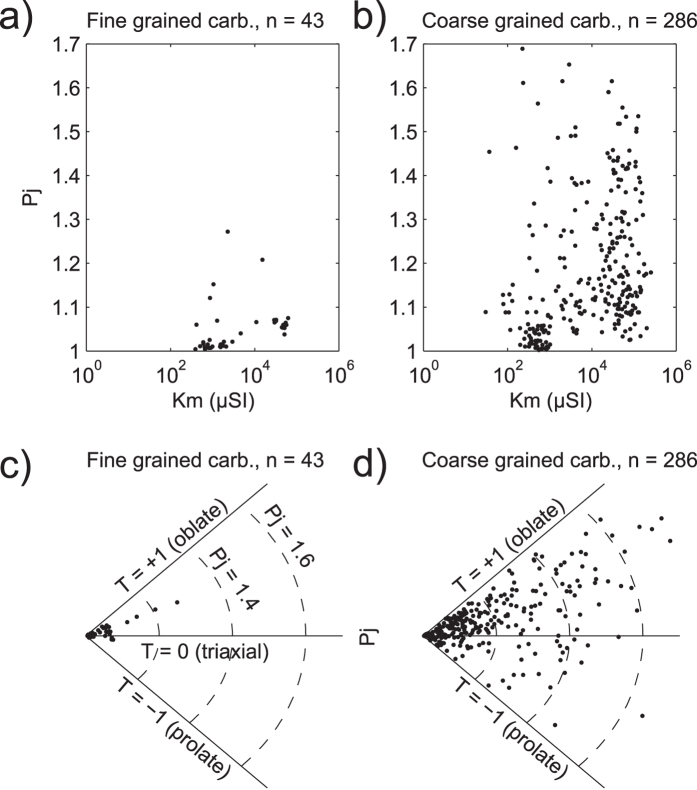
Magnetic susceptibility, degree of anisotropy, and shape factor. (**a,b**) show magnetic susceptibility (*K*_*m*_) versus degree of anisotropy (*P*_*j*_) for fine- and coarse-grained carbonatite samples, respectively. Coarse-grained samples show much higher degree of anisotropy than the fine-grained samples. Carbonatite has a bimodal distribution of *K*_*m*_ (see also Supplementary Figure S1), and a high degree of anisotropy that typically corresponds to strong susceptibility. (**c,d**) show degree of anisotropy (*P*_*j*_) versus shape factor (*T*) for carbonatite samples. All samples are shown in the plots except one triaxial and three oblate carbonatite subsamples, which plot in the *P*_*j*_ range 1.92 – 3.11. The three arcs in each sub-image indicate *P*_*j*_ = 1.2, 1.4, and 1.6, respectively. *P*_*j*_ and *T* are plotted in a polar coordinate system (90° sector)^19^,^20^.

The results of the magnetic field dependent measurements (i.e. hysteresis loops) indicate a mixture of multi- and single-domain states according to available theoretical mixing curves^34^ (Fig. 3d). Multi-domain magnetite has comparatively higher bulk susceptibility than single domain magnetite and the former domain state is likely to control the low-field magnetic anisotropy in carbonatite. The high bulk susceptibility of the samples (>10^−3^ SI) confirms that magnetite dominates the AMS signal (see Supplementary Figure S1). In general, samples with bulk susceptibility lower than 10^−3^ SI have *P*_*j*_ < 1.05, whereas samples with higher bulk susceptibility have *P*_*j*_ values between 1.05 and 1.6 (Fig. 4a,b). Notably, the majority of the AMS data from carbonatites show oblate susceptibility ellipsoids (Fig. 4c,d).

**Figure 5:**
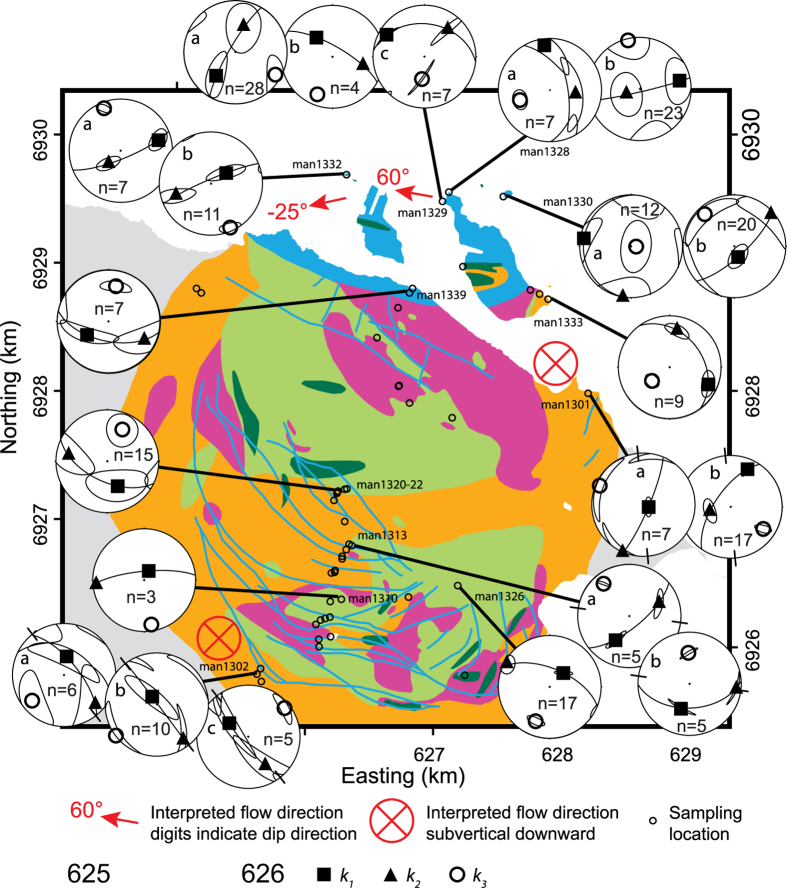
Spatial/geometric results of the AMS measurements. Geological map with lower hemisphere equal-area projections (Schmidt net) for carbonatite samples showing magnetic lineation (squares), foliation (great circles), and the principal axis normal to foliation (*k*_*3*_) as circles. The strikes of the dykes are indicated by two ticks at the rim of each Schmidt net for locations with confident dyke orientation recorded. In the north of the Alnö complex, the magnetic lineations are mostly sub-horizontal, whereas in the south they are mostly sub-vertical. Interpretation of the magma flow in sheets is indicated by red arrows and circles with a cross (indicating plunge and downward flow). Confidence ellipses are given for data groupings of more than five data points. The AMS results are divided in subgroups for those locations with more than one cluster of orientations. For most locations these subgroups are based on the sampling location in the dyke (see Supplementary Table S2). However, for locations man1328 and man1330 the subgroups are based on different orientation of the principle axes. The geological map was kindly provided by © Geological Survey of Sweden. The Schmidt nets were created in the program Anisoft 42 from Agico Inc. (www.agico.com) and overlain on the map in Adobe Illustrator CS4.

Within the Alnö carbonatite sheets, the orientation of the magnetic fabric is generally consistent with the macroscopic foliation recorded in the sheets (Fig. 5), both close to the margin and in the sheet centres. The minimum susceptibility axes (*k*_*3*_) are usually oriented perpendicular to the strike of the Alnö sheet intrusions where it was possible to measure the wall of the dyke, i.e. the magnetic foliation (*k*_*1*_-*k*_*2*_ plane) is sub-parallel to the strike of the sheets. The orientation of the dyke wall was measured at four localities (man1301, man1302, man1329, and man1332) and at three additional localities (man1310, man1313, and man1320–22) the orientations were obtained from earlier regional mapping of these carbonatite sheets^11^,^13^. Maximum principal susceptibility (*k*_*1*_) tends to dip shallow and sub-parallel to the strike of the sheets in the north of the complex, whereas in the south, *k*_*1*_ is often sub-vertical. Also, in the southern part of the complex, the magnetic foliation follows the strike of sheet intrusions, whereas, in the northern part, more variation in magnetic foliation and lineation relative to intrusion strike is observed (Fig. 5, Supplementary Figure S4 and Table S2). The different patterns for the northern and southern areas support earlier studies that indicated that these areas may represent separate pulses or events^13^.

**Figure 6:**
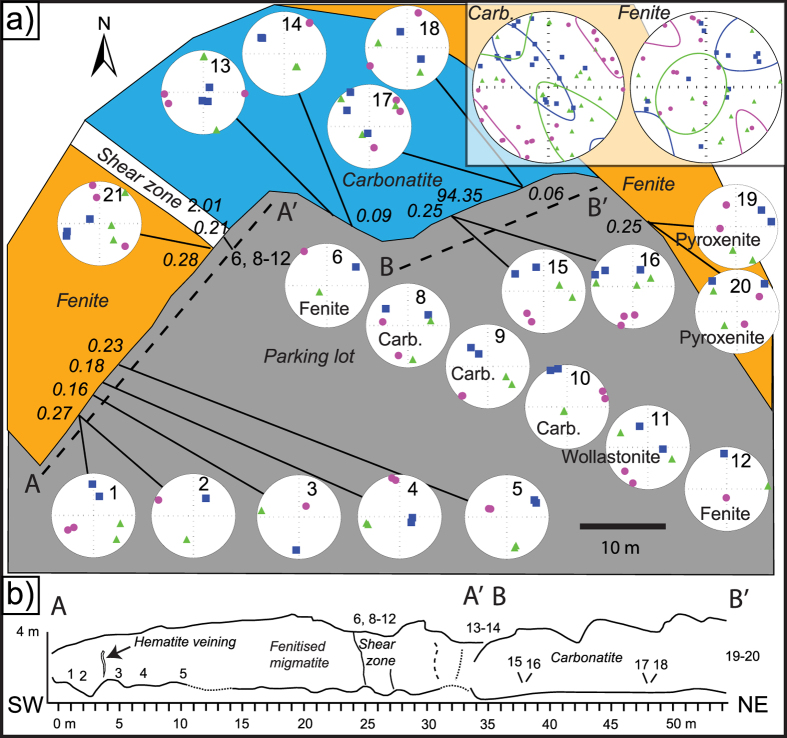
Detailed AMS study of a carbonatite sheet intrusion in the southern part of the Alnö complex. (**a,b**) show map-view and profile-view of the outcrop, respectively. (**a**) Detailed sampling was carried out across a ~25 m wide carbonatite sheet intrusion in Smedgården in the southern part of the Alnö ring complex (Fig. 1). Maximum *k*_*1*_ (squares), intermediate *k*_*2*_ (triangles), and minimum *k*_*3*_ (circles) susceptibility axes are plotted in lower hemisphere equal-area projections (Schmidt net). Numbers in the equal-area nets indicate abbreviated sample numbers. The shear zone in the top left of the map contains wollastonite^14^, while the north-eastern fenite wall rock displays pyroxenite inclusions. The strike of the carbonatite sheet intrusion is NW-SE. Notably, the wall rock (fenitised migmatite) shows greater scattering of *k*_*3*_ than the carbonatite samples (see inset figure in the upper right corner). For carbonatite, *k*_*3*_ concentrates perpendicular to the orientation of the sheet, i.e., the *k*_*1*_-*k*_*2*_ planes follow the sheet plane. The Schmidt nets were created in the program Anisoft 42 from Agico Inc. and overlain on the map in Adobe Illustrator CS4. (**b**) Sketch of outcrop traverse A-A’ and B-B’ from approximately SW to NE with sample locations and field observations marked (based on field data in Skelton *et al*.^14^ and modified in Illustrator CS4).

**Figure 7:**
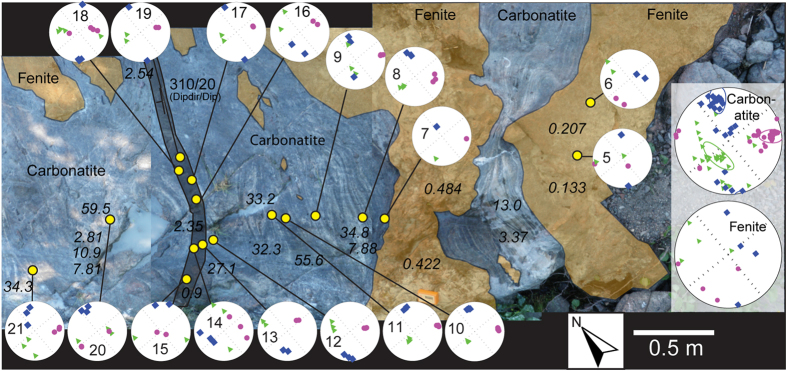
Re-coloured outcrop photograph and AMS results from the eastern part of the Alnö complex. The outcrop (Stornäset harbour, location man1301, see Fig. 1) shows an irregular sheet margin with a general strike in NNE-SSW direction. Pieces of the fenite wall rock have been eroded by, and included into the carbonatite sheet. Pinching of the carbonatite dyke by the fenite wall rock is seen to the right in the image and might be due to sheet closure during the final stage of intrusion. A younger, fine-grained and darker carbonatite dyke cross-cuts the carbonatite sheet intrusion as seen to the left in the image. Maximum *k*_*1*_ (squares), intermediate *k*_*2*_ (triangles), and minimum *k*_*3*_ (circles) susceptibility axes are plotted in lower hemisphere equal-area projections (Schmidt net). Geographic north is indicated in the lower right corner. The italicised numbers indicate the magnetic susceptibility measured by a handheld susceptibility meter (SM-20) in 10^−3^ SI. Numbers in the equal area nets represent abbreviated sample numbers. The inset to the right shows cumulative results of carbonatite and fenite samples in the outcrop. The magnetic foliation in the sheet intrusion follows the sheet orientation. Although there are only four measurements for fenite, it is obvious that *k*_*3*_ values are less tightly distributed in the fenite relative to that in the carbonatite sheet. The thin carbonatite dyke has a magnetic foliation with similar strike compared to the coarse grained carbonatite sheet, but with a more shallow dip. The rock types are coloured in the image to improve the visibility; fenite with sand colour, coarse grained carbonatite in blue colour, and grey colour is used for the late cross-cutting carbonatite dyke.

The minimum susceptibility values (*k*_*3*_) mostly display sub-horizontal orientations, e.g. 62% of *k*_*3*_ dip < 30° and 4.5% dip > 60° for carbonatite. The ellipsoids of most carbonatite samples show oblate shapes (~77%; *T* > 0) and can be visualised as steeply dipping oblate ellipsoids. Despite testing both the *k*_*1*_-method (after^2^) and the two methods that employ *k*_*3*_ (after^5^), it has not been possible to decisively determine a magma flow direction for many of the investigated localities, mainly due to a lack of control from exposed sheet margins (the methods and results are presented in Supplementary Figure S4). We assumed that dyke walls were nearly vertical in these locations when we performed flow calculations. The sites that yielded interpretable results in the south and east (man1301 and man1302) show sub-vertical calculated flow directions with downward direction. In addition, two locations on the small islands in the northern part of the complex (man1329 and man1332) indicate flow directions towards the west, with plunges of 25° upwards and 60° downwards, respectively (Fig. 5). At the two locations in the south and the east of the complex (man1301 and man1302), it was possible to sample carbonatite sheets and their adjacent wall rock (Figs 6 and 7). Both these sheets dip sub-vertical, and the magnetic foliation is sub-parallel to their strike. The principal axes of magnetic anisotropy in the wall rock, in turn, show highly variable orientations that do not conform to the principal axes of the adjacent carbonatite sheet (Figs 6a and 7). This observation cannot be explained by higher bulk susceptibility in carbonatite, because carbonatite bulk susceptibilities are generally low in the southern location, as confirmed both in outcrop with a handheld susceptibility meter (Fig. 6a) and through subsequent laboratory measurements (see Supplementary Figure S2). The AMS measurements of the country rock suite, in turn, do not show any obvious systematic orientation. The fundamentally different magnetic orientations recorded in the intrusive sheets and the country rock consequently implies a different origin for the two rock types.

## Interpreting AMS in Sheet Intrusions

**Figure 8:**
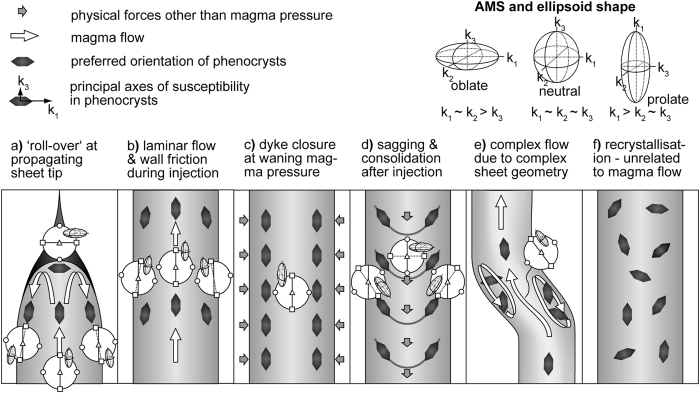
Potential syn- and post-emplacement processes in magmatic sheet intrusions and their expected AMS fabrics. (**a,b,e**) are syn-emplacement processes, while (**c,d,f**) represent post-emplacement processes. The schematic diagram considers endmember scenarios only, but not different ferrimagnetic mineral grain sizes^19^,^66^,^67^,^68^ or the potential interaction of grain-scale magnetic fields^69^,^70^. The relationship between AMS and preferred crystal orientation is so that the crystal long-axis represents *k*_*1*_ and the crystal short-axis represents *k*_*3*_, which is the case for a rock where multi-domain magnetite controls the AMS^3^. Late-stage compaction during dyke closure at waning magma pressure is the most plausible explanation of the magnetic fabric in the investigated Alnö sheet intrusions (see text and Supplementary Figure S5 for details).

The dynamic emplacement of magmatic sheet intrusions is influenced by the complex interaction of fluid- and rock-mechanical parameters, among them magma pressure, host-rock strength, viscous stresses at the magma-rock interface, and the structural configuration of the feeder reservoir and conduit^27^,^33^,^35^. Although cases of sheets showing turbulent flow or post-emplacement deformation have been documented, and thus various complex magma-flow patterns may occur^9^, numerical models suggest that magma flow in sheet intrusions is usually laminar^36^ (Fig. 8). Carbonatite magmas are furthermore considered to have low viscosity. Oldoinyo Lengai’s gas-free carbonatite lavas have apparent viscosities in the range 1 to 5 Pa∙s, more than one order of magnitude lower than those of most basaltic lavas^37^. The low viscosity of carbonatite magma, theoretically, allows turbulence to develop even for relatively low magma flow velocity, yet outcrops in Alnö display ‘flow banding’ that indicates dominantly laminar flow (see Supplementary Figure S3a–c). The northern part of the complex also contains outcrops with crosscutting ‘flow banding’ (see Supplementary Figure S3b), which indicates that the banding is not a result of pervasive post-emplacement recrystallisation. Rotation and alignment of stiffer pyroxenite wall rock fragments are locally observed in carbonatite sheets and show sigma-type clasts (see Supplementary Figure S3c), which is strong evidence for simple shear and hence laminar flow. Although we only observed outcrops that indicate laminar flow, it is conceivable that regimes with turbulent primary flow may have occurred earlier during sheet emplacement. However, it appears that evidence of turbulent flow is not preserved, which must be because either magma viscosity increased during cooling while the flow velocity decreased or because turbulent flow was not a critical factor.

A combined understanding of sheet-emplacement dynamics and the source of the magnetic fabric is crucial in order to employ AMS as a proxy for magma flow directions. Therefore, the multitude of possible interpretations of AMS in sheet intrusions has prompted us to evaluate the available magnetic fabric data in terms of a set of six possible emplacement scenarios. Although individual magnetic fabric studies target one or sometimes more than one of these scenarios (Fig. 8), there is often a lack of a systematic assessment regarding the different emplacement types and their associated magnetic fabrics^18^,^38^,^39^,^40^. Other potential scenarios may exist that are not yet identified here, and notably more than one scenario may be relevant to the same intrusion. Specifically, at the sheet tip, magma flow may resemble lava-flow lobes that produce magnetic fabrics unrelated to the overall magma propagation direction (Fig. 8a, ‘roll-over at propagating sheet tip’; cf.^41^,^42^,^43^,^44^). This rare scenario occurs in places where a sheet or sheet segment terminates, and sampling locations with those settings should be avoided if the overall pattern of the primary flow is of interest. Away from the sheet tip, laminar magma flow during sheet propagation can produce a slight imbrication of phenocrysts, producing magnetic fabrics that reflect magma flow (Fig. 8b, ‘laminar flow plus wall rock friction’; cf.^2^,^10^,^45^). This type of flow imparts a simple shear force that tends to produce a prolate AMS fabric, which has been reproduced both experimentally and numerically^46^. Because inflation of a magmatic sheet must be supported by an influx of more magma, sheet inflation is covered by this scenario too. When sheet geometries are more complex or where sheets exhibit local irregularities, laminar magma flow may be disturbed, and might cause lateral-, helical-, or eddy flow^47^,^48^ (e.g. Fig. 8e, ‘complex flow due to complex sheet geometry’). These emplacement regimes may produce magnetic fabrics that vary across and along a sheet, which make strategic sampling imperative to achieve a representative result^49^. In addition, primary flow fabrics may also be modified by post-emplacement processes, such as sheet closure, e.g. when the width of the sheet decreases once magma pressure is waning (Fig. 8c, ‘sheet closure at waning magma pressure’; cf.^50^,^51^,^52^). As the sheet aperture decreases, the prolate fabric expected from a primary flow-dominated regime would be partially or entirely overprinted by a compaction-related oblate fabric. Another process relevant at this point is when magma in a sheet intrusion starts to solidify along the margins while the sheet centre may still be liquid and sag downward due to progressively lower internal pressure. Sagging can lead to the rotation of phenocrysts and will likely produce a magnetic fabric that strongly deviates from that of primary flow (Fig. 8d, ‘sagging and consolidation after injection’; cf.^53^,^54^). In addition, regional tectonic stresses can affect the original flow fabric at this stage, which would then also be reflected in the AMS fabric^55^,^56^. Lastly, post-emplacement recrystallisation may overprint original flow-related magnetic fabrics and result in the loss of primary magma flow information^19^ (Fig. 8f ‘recrystallisation unrelated to magma flow’). Recrystallisation may overprint AMS fabrics, especially when coupled with a tectonic stress regime and can thus be recognised through a “regional” fabric that is also seen in the sheet intrusions. However, static recrystallisation due to, e.g., initial stages of rock alteration or equilibration on cooling may not affect primary AMS fabric if the mineral arrangement is not significantly disturbed, especially since secondary or later mineral phases tend to mimic the primary mineral fabric^57^,^58^. For the Alnö sheet intrusions we have considered each of the above presented scenarios, and a combination of scenarios, which we now evaluate on the basis of the AMS fabrics and the geologically most plausible emplacement mechanisms.

‘Roll-over at the sheet tip’^41^ is restricted to the propagating end of a sheet. As we have avoided to sample such locations, this scenario can be ruled out for the sheets in our study. Consistency between the magnetic fabric in the centre and towards sheet margins allows us to also rule out a simple ‘sagging and partial backflow after injection’ scenario (Fig. 8d). The absence of consistent AMS patterns between wall rock and carbonatite sheets indicates that tectonic stresses were also not important in influencing the magnetic fabrics after sheet emplacement (Figs 6a and 7). Furthermore, ‘complex flow due to complex sheet geometry’ (Fig. 8e) is likely to occur at a local scale in Alnö, e.g. where a sheet locally deviates from its overall trend. To reduce the likelihood of such complications, we sampled sheet intrusions at least a few metres wide, with locally constant strikes that follow the overall ring-shape of the Alnö complex^11^,^12^,^13^. Large-distance lateral or helical flows (cf.^7^,^48^), although possible, are also not likely for the sheets of the Alnö complex since they are not connected along the circumference of the complex. Pervasive ‘recrystallisation unrelated to magma flow’ can also be excluded because this process would give either a low degree of anisotropy and scattered AMS data or the data would show a regional tectonic stress-induced foliation. The pronounced magnetic foliation and lineation we recorded in the Alnö sheets are not consistent with low degrees of anisotropy and while a recrystallisation under tectonic stress could produce a general regional AMS pattern^59^, no such pattern is identified. Although recrystallisation of calcite has likely occurred (e.g. Fig. 2a–d), the magnetite grains that dominate the magnetic signature most likely did not recrystallise after emplacement of the magma. This is shown by, e.g., the intergrowth of large euhedral magnetite with apatite grains (Fig. 2e), and, in the case of smaller interstitial magnetite, through thin post-formation reaction or recrystallisation rims (e.g. Fig. 2g,h). While post-emplacement recrystallisation of magnetite could influence previously developed fabrics, it seems at Alnö this effect was either happening under static conditions^58^ or was not sufficiently pervasive for magnetite to lose memory of the original fabric. Notably the irregular shape and generally smaller size of late-grown magnetite may result from the competition for space in between earlier formed crystals and thus mimics the macrofabric, while the observed thin rims of approximately 2 μm on magnetite and pyrite grains will have only negligible effects on the AMS signature (Fig. 2f). Furthermore, by growing along the rims of pre-existing mineral grains, recrystallised (secondary) magnetite will also mimic the pattern of the existing minerals and hence their preferred orientation.

The two remaining scenarios, ‘laminar flow plus wall rock friction’ (Fig. 8b) and ‘sheet closure at waning magma pressure’ (Fig. 8c), can explain the observed magnetic fabrics at Alnö. Both processes produce a small angle between the magnetic foliation and the intrusion wall. The calculated flow directions are downward, which is not uncommon in dykes elsewhere (e.g.^9^) and although primary sheet emplacement may involve local lateral and downward flow, the overall magma propagation direction during the initial formation of a cone sheets must be assumed to be upward^28^,^41^. Waning magma pressure and associated sheet closure may hence be the more plausible scenario for the investigated Alnö sheets. This possibility is further supported by the dominant shape of the susceptibility ellipsoids. Figure 4d shows that most carbonatite samples have oblate susceptibility ellipsoids, which requires compression to orient magnetite grains perpendicular to the axis of maximum compaction. Closing and contraction of a sheet after magma injection ceases (Fig. 8c) effectively overprints, in part or in full, any existing primary flow-related AMS signature. It is expected that the overprint causes a change in shape factor (*T*) from a dominantly prolate flow fabric to an oblate compaction-induced secondary fabric that will usually be parallel to the sheet’s wall. Other studies have related the change of the shape factor to syn-emplacement shear stress, for instance close to the Earth’s surface due to near-surface stresses^40^. However, this process should be corroborated by shear stress indicators in the outcrop^39^,^60^, and although we see shear indicators in the Alnö complex in a few locations (see Supplementary Figure S3), the majority of investigated outcrops at Alnö were not in the proximity of the surface^15^ and lack evidence of intense externally-controlled shearing. This observation implies that shear stress probably was not a main control to change the shape factor, leaving late-stage compaction during emplacement as the most plausible fabric-forming process for the sheet intrusions at Alnö.

In order to test the possibility of overprinting (superposition) of the magnetic fabrics, the zone axis method of Henry^61^ was used. This technique compares the intersection of magnetic foliations (zone axes) for all mutual combinations of samples in a population with the distribution of the magnetic lineations in the same population. If the zone axes and the magnetic lineations coincide, it is an indication that more than one magnetic fabric has been recorded in the rock, i.e., overprinting has occurred. The zone axis method was tested at the locations man1301 and man1302 (Supplementary Figure S5), where we had the most data and the best constraints on dyke orientations. The results show that in these locations zone axes and magnetic lineations tend to coincide, which indicates that *k*_*1*_ corresponds to an intersection lineation instead of a mineral orientation. This intersection implies that at least two magnetic fabrics are present in the carbonatites from these locations.

The lesson learnt from our investigation at Alnö is that anisotropy of magnetic susceptibility (AMS) in sheet intrusions can reflect a range of syn- to post-emplacement processes, or a combination of these, and should not be automatically linked to primary magma flow without considering the full range of geologically plausible scenarios. To achieve reliable interpretations from AMS fabrics in sheet intrusions, a combined understanding of magmatic sheet emplacement and the processes that influence magnetic fabrics after primary magma transport is required. Based on the criteria introduced above, we discuss ‘laminar flow plus wall rock friction’ (i.e. syn-emplacement flow) versus ‘sheet closure at waning magma pressure’ and conclude that the AMS fabrics in the Alnö cone sheets became oblate during final sheet closure, because the cone-sheets are discontinuous along strike. Therefore, sheet closure is the most probable process to explain the observed AMS fabrics, and we argue that this pattern is common in cone-sheet fabrics in general, but can be distinguished from, e.g., primary flow through integration of geological, petrological, and AMS data.

## Methods

The sample set used in this study consisted of eight oriented block samples (man101001 – man101008 in Fig. 1 and Supplementary Table S1) collected in 2010 for a test of the applicability of the AMS method and 219 oriented drill cores from 40 separate locations (man1301 – man1342; Fig. 1 and Supplementary Table S1) collected in 2013. The samples for a certain location were named following the convention man130101 (investigator, year, site, sample number). An additional letter at the end (e.g. man130101a) indicates subsamples. The block samples were drilled in a laboratory. In the field, drill cores were collected using a handheld gasoline-powered drill with a diamond-coated drill bit, and were oriented with magnetic and sun compasses.

Most sampling locations lie on an almost north-south oriented transect through the intrusion (Fig. 1). At most locations, two to eight samples were taken, which were then sub-sampled into one to four standard palaeomagnetic specimens (21 mm long and 25.4 mm in diameter)^3^. Six locations were sampled in detail with up to 21 cores, to determine the variation of AMS within outcrops and within individual carbonatite sheets (Supplementary Table S1). Detailed sampling at certain locations provided better statistics for the analysis of the magma flow directions in the sheets. Outcrops studied in detail are situated on the small northern islands and at locations man1301 (on the northern shore of the Alnö Island) and man1302 (in the southern part of the complex) (Fig. 1).

The magnetic susceptibility was measured in 15 different orientations according to the convention illustrated in Jelínek^62^. The drill cores from block samples (collected 2010) were measured with an AC Bridge-device developed by the Geological Survey of Finland, whereas the samples collected in 2013 were measured with an Agico (formerly Geofyzika Brno) KLY-2 Kappabridge. From the results the symmetric second rank tensor, as well as the principal susceptibility axes *k*_*1*_, *k*_*2*_, and *k*_*3*_ could be calculated^62^. The KLY-2 Kappabridge, outfitted with a CS-2 oven, was used to measure susceptibility as a function of temperature on magnetic concentrates extracted from selected carbonatite samples in locations man1301, man1328, and man1333 (Fig. 1). The sample set consisted of small chips (10’s of mg) extracted from carbonatite AMS cores. Continuous measurements were performed while heating the sample up to 700 °C, followed by measurements while the sample cooled gradually down to room temperature. Hysteresis measurements were performed with a M2900-2 alternating gradient magnetometer (Princeton Measurements Corporation) to obtain the saturation magnetisation (*M*_*s*_), saturation remanent magnetisation (*M*_*rs*_), coercivity (*H*_*c*_), and coercivity of remanence (*H*_*cr*_).

Microstructures in a selection of samples were studied in thin sections with standard optical transmission and reflected light microscopy and imaged in backscattered electron microscope using a JEOL JXA-8530F Field electron microprobe at the Centre for Experimental Mineralogy, Petrology and Geochemistry (CEMPEG) laboratory, Uppsala University (^e.g.^ 63,64).

## Additional Information

**How to cite this article**: Andersson, M. *et al*. Magma transport in sheet intrusions of the Alnö carbonatite complex, central Sweden. *Sci. Rep.* **6**, 27635; doi: 10.1038/srep27635 (2016).

## Supplementary Material

Supplementary Information
