# Supplementary material for: Magma transport in sheet intrusions of the Alnö carbonatite complex, central Sweden
Source: Sci Rep. 2016 Jun 10;6:27635. doi: 10.1038/srep27635 (PMC4901264; doi:10.1038/srep27635)
Supplement: Supplementary Information [file srep27635-s1.pdf]

Supplementary Information

Magma transport in sheet intrusions of the Alnö carbonatite complex, central Sweden

Authors: *Magnus Andersson[1], Bjarne S.G. Almqvist[1], Steffi Burchardt[1], Valentin R. Troll[1], Alireza Malehmir[1], Ian Snowball[1], and Lutz Kübler[2]

1: Department of Earth Sciences, Uppsala University, Uppsala, Sweden

2: Geological Survey of Sweden, Uppsala, Sweden

Email address of the corresponding author (M. Andersson): magnus.andersson@geo.uu.se

This file includes Supplementary Figures S1 to S5 and Table S1 to S2.

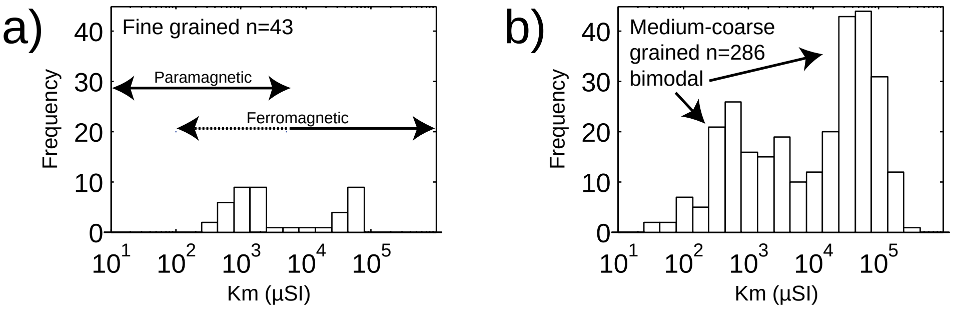

Supplementary Figure S1 | **Bulk magnetic susceptibility.** (a) Fine grained carbonatite and (b) medium-coarse grained carbonatite show bimodality in the distribution of magnetic susceptibility. Number of samples is indicated by n. Susceptibility values above ~2000 µSI are dominated by ferromagnetic (*senso lato*) minerals.

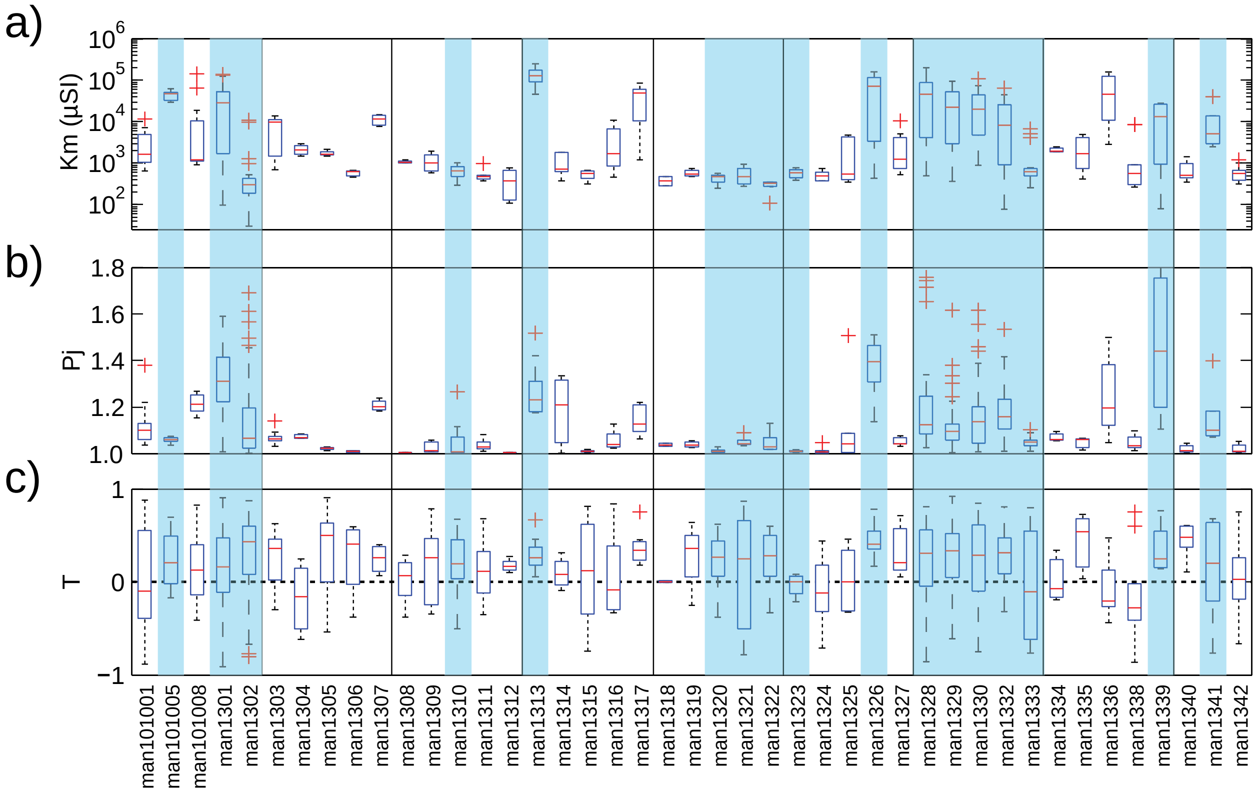

Supplementary Figure S2 | **Boxplot of mean bulk magnetic susceptibility (K_m), degree of anisotropy (P_j) and shape factor (T) for each sampling location.** Locations with carbonatite are marked with blue colour.

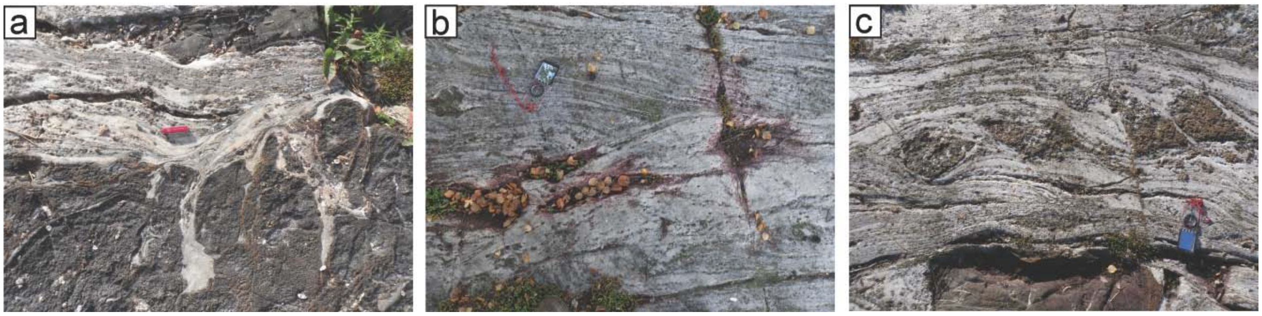

Supplementary Figure S3 | **Typical features visible in outcropping dykes.** (a) Carbonatite dyke injected into fenite wall rock. (b) Carbonatite dyke cross-cutting another carbonatite dyke. (c) Indication of simple shear in a carbonatite dyke is provided by rotation of competent pyroxenite wallrock fragments.

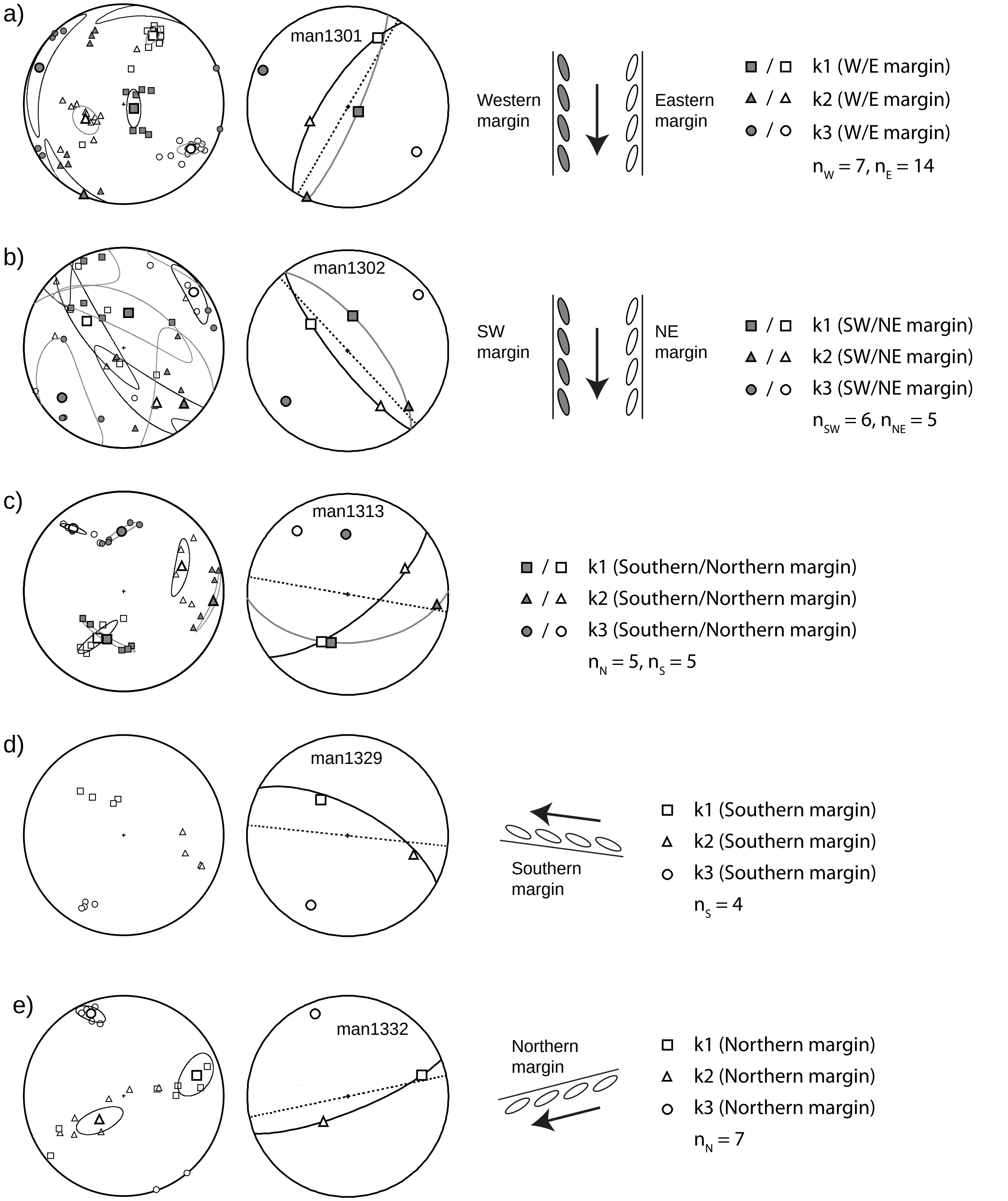

Supplementary Figure S4 | **AMS data and indications of flow directions.** This figure shows the magnetic lineation and foliation of carbonatite samples that were collected close to dyke margins at five locations; man1301, man1302, man1313, man1329, and man1332. For each location two lower hemisphere equal-area projections (Schmidt net) with principal susceptibility axes (k_1, k_2, and k_3; left hand images) as well as their mean axis and confidence ellipses (centre images) are presented. Flow direction is indicated in the

right hand images, except for man1313; for man1301 and man1302 it is a horizontal view along the strike of the dyke and for man1329 and man1332 it is a map view. In the centre part of each subfigure the mean axis and great circles (magnetic foliations) are plotted, as well as a dotted line indicating the attitude of the dyke. In the calculations presented here the assumption with vertical dykes has been made. Although this could only be directly measured in the field for site man1302, it can be inferred that dykes in other locations are very close to vertical (sub vertical). (a) In man1301 the magnetic foliations dip steeply inward at both margins of the dyke. The bisector of the angle between k_3 axes in the opposite margins of the dyke indicates the flow direction; for man1301 that indicates a downward flow. (b) At man1302 a pattern similar to man1301 is observed. Both (a) and (b) may have imbrication that indicates steep downward flow, using the mean flow vector method described by Geoffroy et al.[5]. (c) The method used in (a) and (b) failed for location man1313 as the magnetic foliation has the same orientation at both margins. As only one dyke wall was outcropping in the locations man1329 (d) and man1332 (e), respectively, a method using only one dyke wall was used, as described by Geoffroy et al.(2002)[5]. In the flow calculation the assumption that the dyke was vertical has been made.

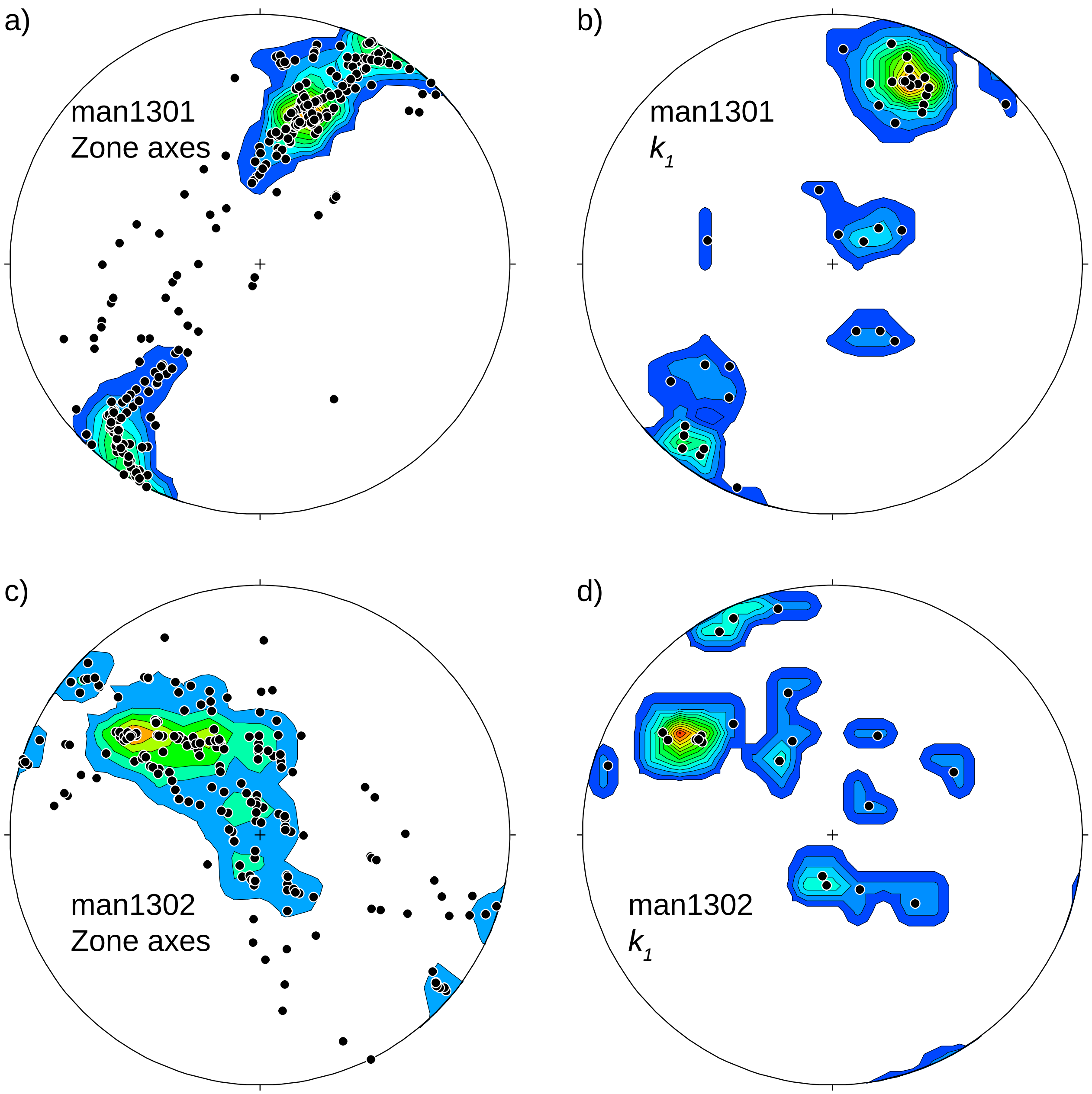

Supplementary Figure S5 | **Zone axes and the principle axes k_1.** In order to test the possibility of overprinting (superposition) of the magnetic fabrics the zone axis method has been used. This technique compares the intersection of magnetic foliations (zone axes) for all mutual combinations of samples in a population with the distribution of the magnetic lineations in the same population. If the zone axes and the magnetic lineations coincide this is an indication that more than one magnetic fabric has been recorded in the rock, i.e. overprinting has occurred. (a) and (b) show the zone axes and k_1, respectively, for carbonatite in location man1301. The majority of the zone axes plot in the same area as most k_1 which indicate that overprinting has occured. (c) and (d) show a similar pattern which indicates that overprinting may have occurred also in location man1302. All the plots are lower hemisphere equal-area projections of a Schmidt net and the contouring is made with counting areas of 1 % of the net area and contour intervals of 2 %.

Supplementary Table S1 | **Oriented rock samples collected on Alnö island.** Some samples were taken as oriented blocks from which cores were drilled in laboratory. These are marked by * in front of n_{cores}. n_{cores}: is the number of cores drilled at a location (except for locations with block samples), $n_{samples}$: is the total number of subsamples from a location, Bt is biotite, and Kfsp is alkali feldspar.

Site	East/North (SWEREF99)	Rock type	n_{cores}	$n_{samples}$
man1301	628209/6927981	Carbonatite, fenite	21	53
man1302	625636/6925776	Carbonatite, fenite, ijolite	21	40
man1303	626113/6926004	Nepheline syenite	4	11
man1304	626108/6926061	Nepheline syenite	2	3
man1305	626086/6926177	Pyroxenite	2	5
man1306	626124/6926211	Fenite	2	5
man1307	626157/6926223	Fenite	2	4
man1308	626195/6926233	Pyroxenite	3	5
man1309	626198/6926356	Ijolite rich in pyroxene	3	8
man1310	626284/6926372	Carbonatite, ijolite	5	11
man1311	626227/6927145	Nepheline syenite	4	7
man1312	626346/6926803	Fenite, ijolite rich in pyroxene	2	4
man1313	626366/6926792	Carbonatite	4	10
man1314	626322/6926761	Nepheline syenite	3	5
man1315	626292/6926707	Nepheline syenite	4	8
man1316	626290/6926689	Nepheline syenite	3	8
man1317	626237/6926599	Nepheline syenite	4	8
man1318	626233/6926585	Nepheline syenite	2	2
man1319	626206/6926578	Nepheline syenite	2	4
man1320	626249/6927199	Carbonatite, carbonatite rich in pyroxene, pyroxenite	3	8
man1321	626256/6927208	Carbonatite rich in pyroxene	2	5
man1322	626258/6927216	Carbonatite rich in Bt	2	5
man1323	626327/6927233	Carbonatite rich in Bt, Kfsp, and pyroxene	3	4
man1324	626312/6927231	Fenite	3	6
man1325	626311/6926980	Nepheline syenite	3	6
man1326	626808/6926391	Carbonatite rich in Bt	6	17
man1327	626727/6928648	Fenite	2	7
man1328	627123/6929551	Carbonatite	15	41
man1329	627073/6929477	Carbonatite	15	49
man1330	627547/6929514	Carbonatite	16	51
man1332	626326/6929686	Carbonatite	8	26
man1333	627898/6928713	Carbonatite (silicosövite)	6	17
man1334	627832/6928756	Ijolite	2	3
man1335	627758/6928785	Pyroxenite, carbonatite	2	3
man1336	627232/6928968	Pyroxenite, ijolite	5	13
man1338	626839/6928797	Fenite	4	10
man1339	626817/6928764	Carbonatite, carbonatite with pyroxene and Bt	3	7
man1340	626563/6928413	Fenite, pyroxenite	4	5
man1341	626729/6928035	Carbonatite, fenite	3	5
man1342	626816/6927904	Fenite, ijolite	6	16
man101001	627149/6927788	Ijolite	*1	12
man101002	625149/6928800	Migmatite	*1	10
man101003	625193/6928764	Fenite	*1	8
man101004	626735/6928040	Nepheline syenite	*1	17
man101005	622539/6929061	Carbonatite	*1	13
man101006	625656/6925799	Carbonatite	*1	8
man101007	625627/6925789	Fenite (quartz-free)	*1	7
man101008	627248/6925781	Pyroxenite	*1	12

Supplementary Table S2 | **Geological and magnetic data summerized for locations with carbonatite.** The data are grouped according to the clustering in Figure 5.

Location	Samples	East/North (SWEREF99)	Rock	Local orient-ation of dyke	Macroscopic foliation	Miscellaneous	Location within dyke
man1301 (a)	1-3	628209/6927981	Carbonatite	030°-210°	Follow local attitude of dyke, winding		Western margin
man1301 (b)	7-12	628209/6927981	Carbonatite, carb. rich in biotite	030°-210°	Follow local attitude of dyke, winding		Eastern margin
man1302 (a)	8-10	625636/6925776	Carbonatite	135°-315° (subvertical)	90°/045° (dip/dip.dir.)		SW margin
man1302 (b)	13-16	625636/6925776	Carbonatite	135°-315° (subvertical)	90°/045° (dip/dip.dir.)		Centre of dyke
man1302 (c)	17-18	625636/6925776	Carbonatite	135°-315° (subvertical)	90°/045° (dip/dip.dir.)		NE margin
man1310	2	626284/6926372	Carbonatite				
man1313 (a)	1-2	626366/6926792	Carbonatite	098°-278°			Northern margin
man1313 (b)	3-4	626366/6926792	Carbonatite	098°-278°			Southern margin
man1320-22	1-2,1-2,1-2	626249/6927199	Carb, carb rich in pyroxene, carb. rich in biotite				
man1326	1-6	626808/6926391	Carbonatite rich in biotite		70°/022° (dip/dip.dir.)		
man1328 (a)	1, 3	627123/6929551	Carbonatite		030°-210° to 060°-240°		
man1328 (b)	2, 4-8, 13-15	627123/6929551	Carbonatite		030°-210° to 060°-240°		
man1329 (a)	6-11, 13-14	627073/6929477	Carbonatite		040°-220° (±10°)	Sinistral sense of shear	
man1329 (b)	1	627073/6929477	Carbonatite	095°-275°	095°-275°		Southern margin
man1329 (c)	12, 15	627073/6929477	Carbonatite		040°-220° (±10°)		
man1330 (a)	10-11, 13, 15-16	627547/6929514	Carbonatite		~90°/022° (dip/dip.dir.)		
man1330 (b)	3-4, 7-8, 12	627547/6929514	Carbonatite		~90°/022° (dip/dip.dir.)		
man1332 (a)	3-4	626326/6929686	Carbonatite	078°-258°	~90°/150° (dip/dip.dir.)	Sinistral sense of shear (see Fig. S3c)	Northern margin
man1332 (b)	5-8	626326/6929686	Carbonatite	078°-258°	~90°/150° (dip/dip.dir.)		
man1333	2-4	627898/6928713	Carbonatite (silicosövite)				
man1339	1-3	626817/6928764	Carbonatite, carb. with pyroxene and biotite				

Supplementary Table S2 continued

Location	N	Kaver (SI)	Pjaver	Taver	K1d/K1i	K2d/K2i	K3d/K3i	K1	K2	K3
man1301 (a)	7	0.11	1.454	0.154	113.1/81.1	203.4/0	293.4/8.9	1.158	0.967	0.876
man1301 (b)	17	0.0449	1.368	0.128	23.7/21.4	258.4/55.9	124.3/25.2	1.132	1.009	0.859
man1302 (a)	6	0.000328	1.132	0.322	7.8/61.2	133.6/17.8	231/21.8	1.026	1.011	0.963
man1302 (b)	10	0.00016	1.275	0.561	327.5/74.7	142.7/15.3	233/1.2	1.052	1.024	0.924
man1302 (c)	5	0.000206	1.875	0.215	305.9/52.6	149.8/35	51.5/11.6	1.133	1.096	0.772
man1310	3	0.000456	1.156	0.318	-	-	-	-	-	-
man1313 (a)	5	0.147	1.298	0.237	209.1/45.6	67.8/37.3	321.4/20.3	1.102	1.021	0.876
man1313 (b)	5	0.125	1.25	0.337	199.1/48.2	97/10.6	358/39.8	1.085	1.023	0.892
man1320-22	15	0.000405	1.039	0.159	157.6/46.7	277.7/25.3	25.2/32.4	1.009	1.003	0.988
man1326	17	0.0604	1.362	0.44	43.8/58.8	297.8/9.5	202.4/29.4	1.117	1.032	0.851
man1328 (a)	7	0.0695	1.093	0.181	353.2/13.4	98.7/48.1	252.2/38.7	1.024	1.012	0.965
man1328 (b)	23	0.0547	1.203	0.311	78.6/22.6	246.8/66.9	346.8/4.2	1.042	1.008	0.951
man1329 (a)	28	0.0332	1.152	0.225	220.2/40.9	17.4/46.8	120/11.6	1.041	1.006	0.953
man1329 (b)	4	0.0666	1.106	0.45	-	-	-	-	-	-
man1329 (c)	7	0.0281	1.043	0.32	299.9/16.5	40.2/31.3	186.2/53.7	1.016	1.003	0.981
man1330 (a)	12	0.00191	1.015	-0.075	279.2/8.1	189.2/0.3	96.8/81.9	1.005	0.999	0.996
man1330 (b)	20	0.036	1.2	0.187	160.5/72.1	49.5/6.6	317.5/16.5	1.067	0.987	0.946
man1332 (a)	7	0.0345	1.213	0.088	74.3/25.1	224/61.5	338.3/12.6	1.074	1.013	0.913
man1332 (b)	11	0.0129	1.204	0.182	54.2/59.7	250.5/29.3	156.5/7.1	1.062	1.019	0.919
man1333	9	0.00062	1.048	-0.203	113.3/19.7	12.3/28	233.6/54.7	1.02	0.999	0.981
man1339	7	0.00776	1.175	0.225	237.1/49	117.5/23.2	12.2/31.6	1.13	1.062	0.808
